# Opioid Sparing Anesthesia and Enhanced Recovery After Surgery Protocol for Pancreaticoduodenectomy

**DOI:** 10.7759/cureus.19558

**Published:** 2021-11-14

**Authors:** Joseph Leech, Kenneth Oswalt, Michelle A Tucci, Oscar A Alam Mendez, Bryan J Hierlmeier

**Affiliations:** 1 Anesthesiology, University of Mississippi Medical Center, Jackson, USA

**Keywords:** opioid-sparing analgesia, eras protocol, ketamine, ketamine infusion, lidocaine infusion, intrathecal opioids, regional blocks

## Abstract

Background

Opioid sparing anesthesia and enhanced recovery after surgery protocols are not innovative ideas. However, the utilization of pancreaticoduodenectomy is limited. With the rise in awareness of the opioid epidemic in the United States, we have created a multimodal approach to anesthesia and postoperative care to limit adverse effects of opioids and curb the use of opioids postoperatively.

Methods

We conducted a retrospective cohort study performed by chart review of an opioid-sparing anesthetic and enhanced recovery after surgery (ERAS) protocol initiated jointly by the anesthesiology departments and transplant surgery for pancreaticoduodenectomy from January 2017 to October 2019.

Results

Demographic data was found to be comparable between the control and protocol groups. Hospital length of stay, ICU length of stay, and opioid requirements significantly decreased in the protocol group. Hospital length of stay decreased from 8.92 to 5.72 days, ICU days decreased from 1.52 to 0.42 days, and narcotics for the first five hospital days were significantly decreased from 130.13 to 71.2 morphine milligram equivalents.

Conclusion

Proper postoperative pain management can improve patient satisfaction and decrease complication rates. Pancreaticoduodenectomy is a complicated procedure with relatively limited data regarding enhanced recovery after surgery protocols. Likewise, there is limited data regarding opioid-sparing anesthesia techniques. Our protocol produced promising hospital length of stay and reduced opioid administration during the first five hospital days without increasing 30-day readmission rates.

## Introduction

Although not a novel concept, Enhanced recovery after surgery (ERAS) programs have proven effective in reducing the surgical stress response leading to improved recovery and decreased hospital cost [1-7]. By standardizing a patient’s perioperative care, a more predictable hospital course with improved outcomes and patient safety may be achieved. Though the benefits of ERAS were initially seen in colorectal surgery, ERAS protocols have been emerging in virtually all surgical subspecialties [1-9]. Despite published guidelines for pancreaticoduodenectomy (PD) by the ERAS society, there is relatively limited published data [10]. It has been suggested that the limited data and implementation of ERAS protocols for PD at institutions is due to the surgical complexity and mortality, and morbidity associated with the procedure [7].

Additionally, there is a presumption that early feeding may lead to increased risk of anastomotic leak and pancreatic fistula formation, despite multiple reports demonstrating safety [11-13]. With medical and surgical advances, perioperative mortality has decreased from 30% to less than 2%, while morbidity has decreased to 30% [14]. The side effects of opioids such as respiratory depression, bradycardia, sedation, delayed emergence, postoperative nausea and vomiting, pruritus, difficulty voiding, and ileus are well known [15-16]. Though morbidity and mortality have seen a decrease, the incidence of chronic opioid use postoperatively has continued to be of significant concern. Limiting exposure to opioids accentuates the use of non-steroidal anti-inflammatory agents, gabapentinoids, regional/neuraxial anesthesia, lidocaine, and ketamine [17]. In an article from the CDC in 2008, it was found that opioid prescription drugs were involved in 73.8% of prescription overdose deaths [18]. Despite a steady decline in opioid prescriptions from 2010-2015, the national opioid-related deaths continue to rise with half of prescription narcotics [19]. Given the use of opioids for post-surgical pain, some patients are thought to be predisposed to develop chronic pain postoperatively [20-22]. With rising awareness of the current opioid epidemic, our department attempted to formulate an opioid-sparing anesthesia and ERAS protocol for PD.

In addition, large bolus dosing with ultra- short-acting opioids, such as remifentanyl, has been shown to increase pain in the perioperative period [23]. Though a multimodal approach to analgesia is a tenet of all ERAS protocols, opioids remain a staple of anesthesia and postoperative analgesia. Literature regarding opioid-sparing anesthesia is lacking; though, some have reported opioid-free anesthetics for those who are morbidly obese to avoid respiratory depression [22]. Given the importance of this multimodal approach, anesthesiologists are essential providers due to their familiarity and expertise with each drug class, regional and neuraxial anesthesia techniques. In collaboration with the hepatobiliary/transplant surgery department, our anesthesia department has implemented a narcotic sparing anesthetic and ERAS protocol for pancreaticoduodenectomy. This retrospective cohort study aims to evaluate the hospital length of stay, intensive care days, and opioid use following the implementation of our protocol. Secondary outcome measures include days to diet advancement and antiemetic use.

## Materials and methods

We conducted a retrospective cohort study comparing patients undergoing PD utilizing opioid-sparing anesthesia and ERAS (Table 1) compared to conventional methods (control).

**Table 1:** Overview of the opioid-sparing anesthesia and enhanced recovery after surgery (ERAS) protocol utilized by the anesthesia department. TOF- Tetralogy of Fallot

Preoperative
Patient Education prior to surgery date in the preoperative clinic
Carbohydrate load 2 hours prior to surgery
Tylenol 1000 mg PO
Gabapentin 300 mg PO
Intraoperative
Neuraxial anesthesia with 200 mcg intrathecal morphine diluted in 2 ml sterile preservative-free normal saline prior to induction
Induction:
Lidocaine 1.5 mg/kg bolus 1 min prior to induction
Ketamine 10 mg on induction
Propofol- 1.5-2.5 mg/kg
Rocuronium 0.6-1.2 mg/kg bolus
Dexamethasone 8 mg IV after induction but prior to incision
Toradol 15 mg after induction
Bilateral transverse abdominis plane blocks using 10 ml 1.33% liposomal bupivacaine diluted with 10 ml of sterile saline each.
Anesthesia Maintenance:
Lidocaine 1.5 mg/kg/hr
Rocuronium re-dose as needed 10 mg at a time titrated to TOF of 1-2
Ketamine: 0.5 mg/kg bolus prior to incision. 10 mg every 30 min if procedure >2 hrs. Stop boluses 60 mins prior to procedure finish.
Postoperative
Ketamine infusion immediately started: 0.15 mg/kg/hr
Lidocaine infusion at 1.5 mg/kg/hr continued from the operating room for 60 minutes
Scheduled Toradol 15 mg q8 hrs x 5 doses
Scheduled Tylenol 500 mg Q8 hrs x 3 days
Neurontin 100 TID
Morphine 1-2 mg is ordered as one-time doses. No PRN orders
Ambulation with physical therapy (PT) assistance morning after the procedure. PT to continue through the hospital stay
Foley to be removed on POD1
NG tube to be removed on POD1. Clear liquid diet to begin the following removal
Drains discontinued when deemed appropriate by surgical team.
Incentive spirometer while in hospital
Discharge
Neurontin 100 TID x 30 days
Tylenol 500 mg PO Q6hrs Scheduled
Colace 100mg BID
Opioid narcotic prescription given for 1 week per primary

Inclusion criteria included age greater than 18 years old and American Society of Anesthesia (ASA) status II-IV and scheduled for an elective PD case from January 2017 to October 2019. Exclusion criteria were age less than 18 years old, ASA status under I or over IV, intraoperative death, surgical case aborted, emergent procedure, or inability to accurately correct data from chart review (Table 2).

**Table 2:** Exclusion and inclusion criteria ASA- American Society of Anesthesiologists

Inclusion Criteria	Exclusion Criteria
At least 18 years old	Age less than 18 years old
Surgical patients posted for elective pancreaticoduodenectomy (whipple) from January 2017 to October 2019	Emergent surgical procedure
ASA 2-4	Surgical case aborted
	Intraoperative death
	ASA <2 or > 4
	Inability to collect adequate accurate data through chart review

The control group consisted of patients with anesthetics and postoperative management for PD before implementing our opioid-sparing protocol. These cases had a multitude of anesthetic techniques, post-operative pain control, and floor management. The majority of patients in this group received both thoracic epidurals and patient control analgesia pumps with morphine or Dilaudid. Occasionally, if pain remained uncontrolled, a ketamine infusion was prescribed in the hospital for up to 72 hours. In the opioid-sparing ERAS group, patients were educated in the preoperative clinic regarding realistic expectations and consumption of a carbohydrate-rich drink 2 hours prior to hospital arrival on the day of surgery. They were given educational handouts and consented to anesthesia. On arrival to the preoperative holding area, protocol patients were given one gram of acetaminophen and 300 mg of gabapentin. They were taken to the operating room, where neuraxial anesthesia was performed with administration of 200-mcg intrathecal morphine diluted into 2 ml of sterile and preservative-free normal saline. Induction of anesthesia was performed with lidocaine (1.5 mg/kg), propofol (1.5-2.5 mg/kg), ketamine (10 mg), and rocuronium (0.6-1.2 mg/kg). Following successful induction and intubation, bilateral transabdominal plane blocks were performed with 10 mL of 1.3% liposomal bupivacaine diluted to 20 ml with sterile normal saline. Antibiotics were administered an infusion of lidocaine was started at 1.5 mg/kg/hr. Prior to the surgical incision, patients were administered 8 mg IV dexamethasone and 15 mg ketorolac. Maintenance of anesthesia was achieved with the volatile anesthetic of choice in addition to ketamine (0.5 mg/kg bolus prior to incision and 10 mg boluses every 30 minutes) and rocuronium (10 mg titrated to train of four monitoring). Postoperatively a ketamine infusion was ordered at 0.15 mg/kg/hr with a linked discontinue order at 72 hours. The lidocaine infusion was also continued for 60 minutes during recovery.

**Figure 1:**
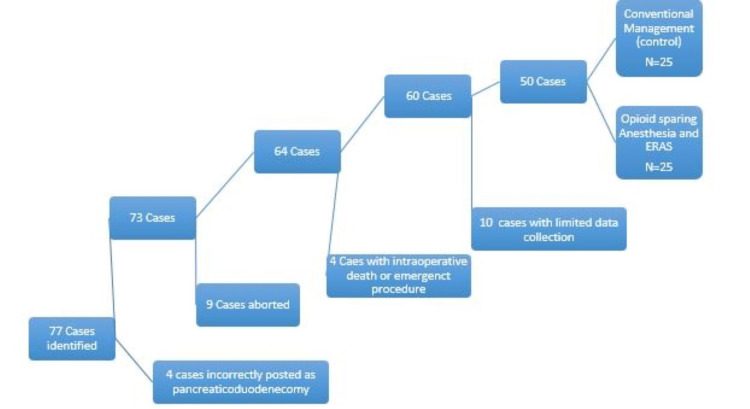
Flow diagram outlining case review and utilization of inclusion/exclusion criteria.

Narcotics were prescribed as a rescue for uncontrolled pain. Ketorolac 15 mg (maximum of 5 doses), acetaminophen 500 mg, and gabapentin 100 mg were scheduled post-operatively by our pain service that managed pain during the initial postoperative period. Surgical drains were removed based on surgeon discretion. Urinary catheter and nasogastric tube were removed on POD 1 if able, followed by a clear liquid diet with advancements as tolerated. Patients were mobilized on the morning of POD1 and continued working with physical therapy during their entire hospital course. Two surgeons posted seventy-seven PD cases in the main operating room from January 2017 to October 2019. Of the 77 cases initially identified via case requests, 27 were omitted due to meeting exclusion criteria or incorrect surgical posting (Figure 1).

Of the 50 remaining cases, 25 were prior to our ERAS protocol being implemented, while the remaining 25 cases were conducted utilizing our narcotic sparing anesthetic and ERAS program. Demographic information including age, sex, body mass index (BMI), American Society of Anesthesiologist classification (ASA), opioid tolerance, and smoking status was evaluated and compared using a student t-test (Table 3).

**Table 3:** Demographic, primary outcome and secondary outcome measures (*p<0.05) MME- morphine milligram equivalents

Variables	Conventional	Narcotic sparing anesthesia and ERAS	P-value
	(Mean ± SD)	(Mean ± SD)	
Age	64.40 ± 4.12	57.88 ± 6.27	0.08
BMI	27.45 ± 2.44	28.58 ± 2.58	0.512
ASA	2.82 ± 0.20	2.72 ± 0.19	0.142
Surgery Time	259.56 ± 28.99	264.40 ± 46.08	0.857
Antiemetic administration	2.84 ± 1.87	2.72 ± 0.19	0.423
ICU length of stay (Days)	1.52 ± 0.922	0.42 ± 0.432	0.042*
Hospital Length of stay (Days)	8.92 ± 2.25	5.72 ± 0.718	0.008*
Time to clear liquid diet (Days)	3.16 ± 1.02	1.76 ± 0.43	0.012*
Time to regular diet (Days)	5.18 ± 1.54	3.20 ± 0.50	0.017*
Intraoperative morphine milligram equivalents (MME)	33.29 ± 6.39	2.6 ± 2.53	<0.001*
MME during POD 0-5 (- intraop MME)	130.13 ± 52.37	71.32 ± 30.13	0.004*
MME cumulative (intra + POD 0-5)	162.09 ± 52.14	73.92 ± 29.98	0.004*

The Chi-square test was utilized for statistical analysis of primary and secondary outcomes (Table 3). Primary measurement outcomes included hospital length of stay and total narcotic usage in morphine equivalents (MME) for the postoperative days (POD) 0-5. Secondary outcomes include time to diet advancement, intensive care days, antiemetic usage during POD 0-5, and 30-day readmission. P values less than 0.05 were taken as statistically significant.

## Results

Demographic data regarding age, sex, BMI, and ASA status were statistically insignificant (p-value > 0.05) when groups were compared using the student t-test. Surgical time was also found to be statistically insignificant in the control group (259.56 minutes) versus the protocol group (264.4 minutes) (p-value 0.857). Hospital length of stay, ICU length of stay, and opioid requirements significantly decreased in the protocol group. In the narcotic sparing group, hospital length of stay was decreased from 8.92 days to 5.72 days, a 3.2-day decrease compared to the control group. The number of ICU days were also decreased from 1.52 to 0.42 days on average (p-value of 0.042). Opiate use was significantly reduced (p-value 0.004) for the first five hospital days from 130.13 MME to 71.32 MME, a reduction of 45%. When intraoperative narcotics were incorporated in the data, MME was reduced from 162.09 to 73.92, a total reduction in narcotic use in MME of 54% for hospital days 0-5. Surprisingly, antiemetic use was not significantly improved in the protocol group (p-value 0.423). However, the time to clear liquid diet (CLD) and regular diet were reduced from 3.16 and 5.18 to 1.76 and 3.20, respectively (p-values <0.05).

## Discussion

ERAS protocols attempt to improve patient care, limit error, and provide a predictable hospital course by utilizing evidence-based practice and standardization of care. Based upon ERAS published guidelines, we developed an ERAS protocol that focused on patient education, nutrition, and multimodal analgesics to reduce opioid consumption and improve outcomes. Like other ERAS protocols, we began our standardization in the preoperative clinic, educating patients on expectations and realistic goals of care and pain to educate and limit anxiety. In other surgical specialties, such as orthopedic surgery, patient education was found to positively impact both postoperative narcotic usage and earlier cessation of narcotic following discharge [24]. Others suggest an educational component decreases anxiety, improves recovery, and faster hospital discharge [25-29]. To standardize for nutritional variations, prior to implementing our protocol, patients were made nil per os (NPO) at a minimum of 8 hours prior to surgery and most commonly at midnight the day before regardless of surgical timing. However, fasting has been shown to generate a catabolic state resulting in lower glycogen stores, increased insulin resistance, and increased need for antiemetics postoperatively [30-33]. We chose to utilize a carbohydrate-rich drink 2 hours prior to surgery that has been shown to decrease insulin resistance and nausea/vomiting without increased risk for aspiration [30-34]. In order to address proper utilization of analgesics and anesthesia for PD patients, we based our selection of agents and anesthesia techniques on randomized clinical trials that showed efficacy in reducing opioid use. Randomized control trials have confirmed the efficacy of pain control with oral acetaminophen [35], though a relatively weak analgesic, a synergistic effect occurs when combined with NSAIDs has been suggested [36]. Ketorolac is a COX-1 inhibiting NSAID, which alone has been found to decrease postoperative narcotic use by 25-45% [37-39]. We chose to use the NSAID ketorolac in addition to preoperative oral acetaminophen to exploit their narcotic sparing, analgesic, and anti-inflammatory effects. Likewise, gabapentin has been shown to prevent hyperalgesia through its effect on the dorsal horn ganglion [40-41]. Though no studies were found specifically for pancreatic surgery, gabapentin has been shown by multiple studies to be safe and reduce morphine consumption post operatively in other types of surgery [42-43]. In addition to oral and intravenous pain medications, regional and neuraxial anesthesia techniques have also demonstrated the ability to reduce pain in the perioperative period while minimizing side effects, improving post-operative ambulation, and providing quicker times to bowel function [15].

Epidural catheters are frequently utilized, but removal may become an obstacle in some cases due to anticoagulation and postoperative coagulopathy in hepatobiliary surgery [44]. Likewise, epidural analgesia may impede physical therapy postoperatively due to weakness. Possible complications of epidural administration include inappropriate drug infusion, risk of infection, and failure to achieve adequate analgesia due to catheter migration, dislodgement, and kinking [45-48]. In a 2006 study, preoperative spinal anesthesia with intrathecal morphine was equally effective to an epidural technique in pain control for the first 48 hours following liver resection when used with other adjunctive pain medications [49]. Prior studies have suggested the ideal dose of intrathecal morphine between 100-200 mcg, while doses above are not recommended [50-56]. Additionally, transversus abdominis plane blocks have been shown to reduce opioid requirements after midline abdominal surgery for the first 24hrs and up to 72 hours when liposomal bupivacaine is utilized [57-58]. Before incision, we utilized both spinal and transverse abdominal plane blocks to contest both somatic and visceral pain nociception. Lidocaine and ketamine infusions have also shown promise for pain management in the perioperative period. In a meta-analysis performed by Market et al., operative and postoperative low dose lidocaine infusion was found to reduce postoperative ileus, nausea, and vomiting, hospital stay, and pain [59-64].

Lidocaine infusion, at dosages between 1.5- 3 mg/hr, has been shown to reduce postoperative pain scores in open and laparoscopic abdominal surgery [63-66]. Similarly, low dose ketamine infusions have been shown to decrease narcotic use post-operatively through the first 48 h [66-69]. Our multimodal opioid-sparing anesthetic and ERAS protocol was found to impact multiple primary and secondary outcome measures significantly, as shown in Table 3. Primary outcome measures such as hospital and ICU length of stay showed a capacity for improved patient care and an opportunity for financial impact for both the patient and institution. Though additional procedures were required for our protocol, surgical times were not significantly affected (p-value 0.857). Moreover, our data suggest no significant difference in 30-day readmission rates, which implies non-inferiority to safety compared to conventional care. Surprisingly, despite a significant decrease in postoperative MME, there was no significant difference found in antiemetic use between the two groups suggesting a more complex multifactorial etiology for associated nausea and vomiting. Though anti-emetics use was equivocal, diet advancement was significantly improved with a clear liquid diet (CLD) beginning POD 1.76 and a regular diet usually beginning on POD 3.2. In contrast, the conventional group began CLD on POD 3.16 (Table 3). It is unclear whether quicker diet advancement leads to increased nausea and vomiting, negating any antiemetic benefit produced by our interventions. We find our study very promising for the future of opioid-sparing anesthesia and ERAS protocols; however, we are not without significant limitations. We were unable to collect accurate pain scores during our data collection secondary to inconsistencies in nurse documentation. Earlier discharge from the hospital would indicate that adequate pain control was reached earlier; however, there is a lack of documented data and a potential for bias. Post-discharge pain medication use was also not evaluated or measured in this study. Additionally, given the multitude of interventions and practices employed, we cannot determine essential and unnecessary aspects of our team’s protocol.

Our study demonstrates an equally safe and successful approach to narcotic sparing anesthesia and ERAS for PD as compared to conventional measures. Larger prospective clinical trials assessing individual interventions are needed at multiple institutions to determine the overall effectiveness of our protocol.

## Conclusions

Proper postoperative pain management improves patient satisfaction scores, but more importantly, it leads to early mobilization, reduced risk of thromboembolism, enhanced recovery, early bowel function, and decreased hospital expenditures. We devised an opioid-sparing anesthetic and ERAS protocol utilizing evidence-based medicine to help combat the current opioid epidemic. Our retrospective cohort study focusing on PD demonstrates that narcotic sparing anesthesia and ERAS can effectively limit narcotic use while simultaneously decreasing hospital stay, ICU length of stay, and time to diet advancement. Larger prospective studies are needed at multiple institutions to confirm and expand on our findings.
